# Evidence Clearinghouses as Tools to Advance Health Equity: What We Know from a Systematic Scan

**DOI:** 10.1007/s11121-023-01511-7

**Published:** 2023-03-01

**Authors:** Bomi Kim Hirsch, Michael C. Stevenson, Marjory L. Givens

**Affiliations:** University of Wisconsin Population Health Institute, University of Wisconsin–Madison, Madison, WI USA

**Keywords:** Evidence clearinghouse, Evidence-based program registries, Health equity impact, Health disparity impact

## Abstract

Evidence clearinghouses evaluate and summarize literature to help decision-makers prioritize and invest in evidence-informed interventions. Clearinghouses and related practice-oriented tools are continuously evolving; however, it is unclear the extent to which these tools assess and summarize evidence describing an intervention’s impact on health equity. We conducted a systematic scan to explore how clearinghouses communicated an intervention’s equity impact and reviewed their underlying methods and how they defined and operationalized health equity. In 2021, we identified 18 clearinghouses that were US-focused, web-based registries of interventions that assigned an intervention effectiveness rating for improving community health and the social determinants of health. We reviewed each clearinghouse’s website and collected publicly available information about their health equity impact review, review methods, and health equity definitions and values. We conducted a comparative analysis among select clearinghouses using qualitative methods. Among the 18 clearinghouses, fewer than half (only seven) summarized an intervention’s potential impact on health equity. Overall, those seven clearinghouses defined and operationalized equity differently, and most lacked transparency in their review methods. Clearinghouses used one or more approaches to communicate findings from their review: summarize study findings on differential impact for subpopulations, curate interventions that reduce health disparities, and/or assign a disparity/equity rating to each intervention. Evidence clearinghouses can enhance equity-focused methods and be transparent in their underlying values to better support the uptake and implementation of evidence-informed interventions to advance health equity. However, clearinghouses are unable to do so without underlying equity-focused empirical evidence.

**Supplementary Information:**

The online version contains supplementary material available at 10.1007/s11121-023-01511-7.

Evidence clearinghouses (henceforth “clearinghouses”), also called evidence-based program registries, systematically conduct reviews of evidence to determine the effectiveness of an intervention (i.e., policy, program, or practice) (Horne, 2017; Means et al., 2015). Clearinghouses compile research findings about an intervention, assess the quality and strength of the available evidence, and summarize these findings (Mayo-Wilson et al., 2022; Paulsell et al., 2017). Clearinghouses serve as research dissemination channels that can assist practitioners, including policy makers, grant writers, advocacy groups, and administrators (Burkhardt et al., 2015; Paulsell et al., 2017), who often have limited time or access to scientific literature (Brownson et al., 2018). Clearinghouses advance evidence-based interventions by encouraging the adoption of effective interventions and discouraging the adoption of interventions that have shown negative effects or have not been well studied (Buckley et al., 2022).

Fields like medicine have used evidence-based interventions for centuries (Claridge & Fabian, 2005; Mackey & Bassendowski, 2017). Although scientific research methods have been used to develop evidence-based interventions since the 1960s (Means et al., 2015), growing attention was placed on the use of evidence-based interventions to address health and social issues by the federal government in the late 2000s (Stack, 2018). Federal recognition of the importance of evidence-based interventions led most government agencies to primarily fund interventions that have evidence demonstrating their effectiveness (Burkhardt et al., 2015; Mayo-Wilson et al., 2022; Means et al., 2015). Federal agencies host clearinghouses in areas of education, criminal justice, social welfare, and health to directly link research to funding decisions (Paulsell et al., 2017). Federal clearinghouses improve the visibility of interventions which may also inform research funding and future direction (Burkhardt et al., 2015). Nonprofit organizations and academic institutions also host clearinghouses.

Clearinghouses vary in how they identify which interventions to include in a registry, compile literature, determine causality, and assess program effectiveness (Bergum et al., 2019; Burkhardt et al., 2015; Paulsell et al., 2017). For federal clearinghouses, the purpose, types of interventions included, and the sources reviewed are often defined in the legislation establishing the clearinghouse. Further, clearinghouses provide varying levels of context-specific information to help users determine if an intervention could be effective in their setting, with most clearinghouses providing information about participants or target populations and fewer describing context about implementation fidelity, the implementing agency, costs, or staffing (Horne, 2017). Clearinghouses also vary in how they address an intervention’s readiness to be disseminated, with few clearinghouses including the availability of intervention materials, trainings, and guidance on how users can adopt the intervention for their community while maintaining fidelity (Buckley et al., 2020; Paulsell et al., 2017). Variability in clearinghouses’ methods and approaches means that findings from clearinghouses are not always comparable to one another (Means et al., 2015).

## Health Equity, Inequities, Disparities, and the Social Determinants of Health

Health equity researchers and practitioners often use related terms like equity, inequities, disparities, and the social determinants of health. We refer to Braveman et al. (2017) definitions of equity and disparities for this study because they are widely used in public health. Braveman et al. (2017) define health equity as “everyone has a fair and just opportunity to be as healthy as possible” (p. 2). Health disparities are a way to measure progress toward advancing health equity, and Braveman et al. (2017) further describe the relationship between health equity and disparities as “health equity means reducing and ultimately eliminating disparities in health and its determinants that adversely affect excluded or marginalized groups” (p. 2).

The social determinants of health are the social and economic factors, including education, income, and housing, that influence health outcomes (Braveman et al., 2011; Commission on Social Determinants of Health [CSDH], 2008), and the uneven distribution of these determinants contributes to wide health disparities and inequities across populations (CSDH, 2008; National Academies of Sciences, Engineering, and Medicine [NASEM], 2017a). Addressing disparities and inequities in the social determinants of health can affect health disparities and inequities (Solar & Irwin, 2010). Many clearinghouses compile interventions that address the social determinants of health, such as job opportunities, healthy behaviors, safe and clean environments, civic engagement, health care, and community programs.

## Clearinghouses’ Role in Advancing Health Equity

Clearinghouses help practitioners identify which interventions may result in improved outcomes for an overall community or target population; however, it is unclear the extent to which they have invested in assessing an intervention’s impact on health equity within or between populations. There are several potential ways clearinghouses can evaluate and summarize evidence to help practitioners understand how an intervention can advance health equity. For example, clearinghouses can provide information about an intervention’s differential impact — how an intervention benefits some groups or areas more than others (Milton et al., 2011) — to help users better understand population groups that can benefit from interventions, and whether interventions impact disparities between subgroups. Universal interventions, also called population-level interventions, can generate differential impact (Milton et al., 2011). Interventions that can improve health outcomes on average do not necessarily have a positive impact on health equity. In fact, sometimes, interventions even worsen health disparities and inequities (Jansen et al., 2022). Researchers acknowledge the importance of assessing interventions’ differential impact (Jaciw, 2020; Maden, 2016; Van Horn et al., 2015), but there is still limited empirical research measuring differential impacts which limits how clearinghouses assess differences in effectiveness among subgroups. For example, a recent systematic review of systematic reviews on adolescent population health interventions found limited published evidence about differential impacts (Macintyre et al., 2020).

Clearinghouses can also help practitioners consider the level at which an intervention is working and its potential to change underlying systems that sustain inequity. For example, many interventions focus on the individual or interpersonal level which may not address the environmental and system-level factors that are needed to sustain improvements and address underlying determinants of health (Brown et al., 2019; Thornton et al., 2016). Clearinghouses can also help practitioners focus attention on various social identities within and among population groups (e.g., place of residence, race/ethnicity, occupation, gender, religion, education) (Higgins et al., 2022; O’Neill et al., 2014) and guide them to understand how an intervention may benefit or harm one group more than another.

To our knowledge, no studies have examined how and the extent to which clearinghouses disseminate information on an intervention’s potential impact on health equity. In this study, we conducted a systematic scan to explore the state of the field for clearinghouses.

## Methods

We created a comprehensive directory of clearinghouses following the search methods of Bergum et al. (2019). We identified clearinghouses through an internet search using terms such as “evidence clearinghouse,” “evidence-based program registries,” and “evidence rating.” We cross-checked the list with clearinghouse directories collated by organizations such as the Pew Charitable Trusts, AmeriCorps, health.gov, and the Office of Planning, Research & Evaluation.

**Fig. 1:**
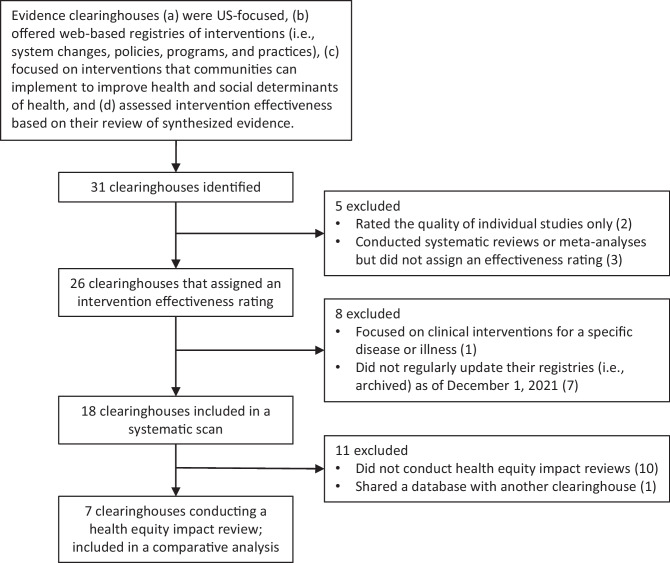
A flowchart of clearinghouse identification and inclusion. Note: five clearinghouses were excluded because they rated the quality of individual studies only (healthevidence.org and Strengthening Families Evidence Review Clearinghouse for Labor Evaluation and Research (CLEAR)) or conducted systematic reviews but did not assign an effectiveness rating (Campbell systematic reviews, Cochrane systematic reviews, and Best Evidence Encyclopedia). Seven evidence clearinghouses that had archived registries as of December 1, 2021 (i.e., Agency for Healthcare Research and Quality Innovation Exchange, Health Care Innovations Exchange, Suicide Prevention Resource Center’s (SPRC) Evidence-Based Prevention, Teen Pregnancy Prevention (TPP) Evidence Review, the Coalition for Evidence-Based Policy (Social Programs that Work), the Promising Practices Network, and 100 Million Healthier Lives Change Library: Bright Spots) and one clearinghouse that focused on clinical interventions for a specific disease (i.e., Compendium of Evidence-Based Interventions and Best Practices for HIV Prevention) were additionally excluded from the systematic scan

We included clearinghouses that were US-focused, offered web-based registries of interventions (i.e., system changes, policies, programs, and practices), focused on interventions that communities can implement to improve health and the social determinants of health, and assigned an intervention effectiveness rating based on their review of synthesized evidence. We excluded clearinghouses from our analytic review if they (a) rated the quality of individual studies only, not the intervention; (b) conducted systematic reviews or meta-analyses but did not assign an effectiveness rating; (c) focused only on clinical interventions for a specific disease or illness; or (d) did not regularly update their registries as of December 1, 2021. Figure 1 shows clearinghouse identification, inclusion, and exclusion criteria for this study.

For the identified clearinghouses, a lead researcher collected publicly available information on how clearinghouses conducted health equity impact reviews from each clearinghouse’s website as of December 1, 2021.^1^ The data was analyzed independently by two researchers who then interpreted the results together. We broadly defined health equity impact review to include various approaches clearinghouses might use, including presentations of an intervention’s differential impact, assessments of an intervention’s ability to reduce existing disparities in health and the social determinants of health, assessments of whether an intervention is equitably designed and implemented, and assessments of how an intervention enhances opportunities for subpopulations and makes systems that affect health and the social determinants of health more equitable. We also collected characteristics about clearinghouses including topic areas covered, host organization, and availability of search functions that allow users to filter interventions by specific health equity subpopulations or topic areas.

We conducted a comparative analysis of clearinghouses with publicly available health equity impact reviews on their websites. Our analysis explored how clearinghouses communicated an intervention’s health equity impact and reviewed their health equity definition and underlying methods. The analysis was guided by these key questions: (1) how does the clearinghouse define equity, and how does the definition guide their health equity impact review? (2) Does the clearinghouse assess the impact an intervention has on disparities, equity, or both? (3) How does the clearinghouse review an intervention’s health equity impact and present results? (4) Are the health equity impact review methods publicly available? (5) What population groups (e.g., racial, gender, geography) does the clearinghouse consider when examining equity impact? (6) What equity-related intervention characteristics (e.g., intervention level — individuals, communities, and systems change; targeting approach — universal, targeted, and combined) does the clearinghouse consider in the health equity impact review?

## Results

We identified 18 clearinghouses that assigned an intervention effectiveness rating. Ten clearinghouses were hosted by governmental agencies, and eight were hosted by academic institutions or nonprofit organizations (see Supplementary material). Most clearinghouses (*N* = 14) provided search filters to identify interventions for populations experiencing disadvantages or interventions aiming for an equity-enhancing goal, such as target population (e.g., racial or age group) or setting (e.g., rural) (see Supplementary material). However, most clearinghouses (61%) did not assess evidence for an intervention’s impact on existing health disparities or inequity. Generally, clearinghouses assessed an intervention’s effectiveness based on average effects and did not consider differential impacts across population groups. Most clearinghouses did not consider how an intervention contributes to worsening, sustaining, or reducing existing disparities or inequities. Only seven of the 18 clearinghouses conducted health equity impact reviews: Blueprints for Healthy Youth Development (Blueprints), California Evidence-Based Clearinghouse for Child Welfare (CEBC), CrimeSolutions,^2^ Home Visiting Evidence of Effectiveness (HomVEE), Maternal and Child Health (MCH) Innovations Database (limited to the content published after MCH Innovation Hub was redesigned in May 2021), The Guide to Community Preventive Services (The Community Guide), and What Works for Health (WWFH). In the following sections, we present our in-depth analyses of the seven clearinghouses.

### Clearinghouse Definitions of Equity

We compared how all 18 clearinghouses defined or operationalized equity by analyzing their vision and mission statements as well as any public information about equity or similar concepts on the clearinghouse and its host organization’s webpages. Eleven clearinghouses that did not conduct a health equity impact review also did not provide any explicit definitions or a vision statement about equity. Among the seven clearinghouses conducting a health equity impact review, three clearinghouses (The Community Guide, WWFH, and MCH) explicitly connected health equity to equal and just opportunities to be healthy. Two (WWFH and MCH) provided a clear definition of health equity and stated that health equity is an organizational core value via their vision statement. Two clearinghouses (The Community Guide and WWFH) addressed the social determinants of health to explain what causes health disparities. One clearinghouse (CEBC) acknowledged existing disparities with discussion about potential effects of biases and societal factors on such disparities but did not provide a clear definition of equity nor a vision statement. Three clearinghouses (Blueprints, CrimeSolutions, and HomVEE) did not provide any explicit definitions or a vision statement about equity (see Table 1).

**Table 1:** Health equity impact review approaches in selected clearinghouses

	Blueprints for Healthy Youth Development		CrimeSolutions	California Evidence-Based Clearinghouse for Child Welfare	Home Visiting Evidence of Effectiveness (HomVEE)	The Community Guide	What Works for Health	MCH Innovations Database
Defines equity	No		No	No	No	Yes (no citation)	Yes (Braveman 2017)	Yes (Braveman 2017)
Focus of review	Disparities in intervention outcomes		Disparities in intervention outcomes	Disparities in intervention outcomes and equity in implementation	Disparities in intervention outcomes	Disparities in intervention outcomes	Disparities in intervention outcomes	Disparities in intervention outcomes and equity in implementation
Population groups of interest	Age groups Race/ethnicity groups Socioeconomic status		Age groups Race/ethnicity groups Socioeconomic status Urbanization levels Gender groups Family structure types	Race/ethnicity groups	Race/ethnicity groups Parent’s mental health or abuse history in childhood Caregiver types ^a^	Age groups Race/ethnicity groups Socioeconomic status (income) Urbanization levels Gender groups	Race/ethnicity groups Socioeconomic status Urbanization levels	Race/ethnicity groups Socioeconomic status Urbanization levels
Intervention level of interest	Individual Family Community		Individual Family Community	Individual Family Agency	Family	Community System	Individual Family Community System	Individual Family Community System
Intervention’s targeting approach	Targeted Universal		Targeted	Targeted	Targeted	Targeted Universal	Targeted Universal	Targeted Universal

		Approaches to presenting health equity impact review						
Summarize study findings on intervention impact across population groups	Differential impact between and within study populations (including subgroup analysis)		Differential impact between and within study populations (including subgroup analysis)	No	Differential impact within study populations (limited to findings of replicated subgroups)	Differential impact between and within study populations (including subgroup analysis)	Differential impact between and within study populations (including subgroup analysis)	Differential impact between and within study populations (including subgroup analysis)
Curate interventions that address disparities or inequity	No		No	Curate a list of 13 child welfare programs that reduce disparities and disproportionality	No	Curate a list of 12 policies and programs in education and housing that reduce health inequity ^b^	No	No
Rate each intervention based on the assessment of health equity impact	No		No	No	No	No	Assign a disparity rating to each intervention ^c^	Assign a rating designation to each intervention ^d^
Where the review and analysis is presented	A designated section on an individual intervention page: “Racial/Ethnicity/ Gender Details”		A designated section on an individual intervention page: “Other information (Including Subgroup Findings)”	A featured topic page: “Reducing racial disparity and disproportionality in child welfare”	A designated section on a model summary brief page A table of study characteristics on the details page of each manuscript	A featured topic page: “Health equity” A designated section on an individual intervention page: “Health equity and education”	A designated section on an individual intervention page: “Impact on disparities” Subgroup findings are presented in “Evidence of effectiveness” ^e^	A designated section on an individual intervention report: “Health equity”

^a^HomVEE’s subgroup information is limited as it started to report such information in 2021. This may not cover all subgroups of interest

^b^At the time of manuscript submission, The Community Guide is updating the health equity content on their website

^c^Three rating categories: likely to decrease disparities, likely to increase disparities, no impact on disparities likely

^d^Four rating designation categories: cutting-edge, emerging, promising, best

^e^What Works for Health is working on adding an equity analysis section on each intervention’s page

### Focus on Disparities in Outcomes vs. Equity in Design and Implementation

Interventions can be reviewed for impacts on disparities in their outcomes (e.g., reduced disparities in access to affordable housing between racial groups), equity in their design and implementation (e.g., equitable design and implementation in housing programs), or both. Five of the seven clearinghouses examined an intervention’s impact on disparities in health outcomes and relevant social determinants of health (Blueprints, CrimeSolutions, HomVEE, The Community Guide, and WWFH). Other clearinghouses examined both disparities in outcomes and equity in implementation. CEBC focused on an intervention’s potential impact on racial outcomes and equity in implementation among families, staff, and agencies engaged in the decision-making process in the child welfare system. MCH also examined both disparities in outcomes and equity in implementation. For example, MCH assessed how equitably practices were performed among staff and stakeholders in program design, delivery, and implementation.

### Presentation of Health Equity Impact Review Findings

We identified three distinct, but not exclusive approaches clearinghouses used to communicate health equity impact review findings. One approach used by clearinghouses was to summarize study findings on differential impact, including subgroup analysis findings for subsets of participants within a study or for subsets of studies with different study characteristics (e.g., effectiveness of an intervention implemented in different geographical locations). A second approach was to curate a list of interventions that could reduce disparities or enhance equity. A third approach was to assign a disparity/equity rating to each intervention.

All seven clearinghouses used at least one of these approaches (Table 1). Blueprints, CrimeSolutions, and HomVEE summarized subgroup analysis findings. Blueprints provided research findings related to differences in intervention effects across subgroups and among various social identities (e.g., “Study 1 found the intervention effect on intention to refuse substances was stronger for female students than male students”). CrimeSolutions reported subgroup effects by broad types of characteristics of participants and programs, such as participants’ social status, criminal record, program activities, and program completion status. HomVEE also reviewed subgroup findings by broad types of participant and program characteristics, such as maternal psychological resources level. However, it only reported subgroup findings replicated in distinct samples.^3^

CEBC and The Community Guide curated a subset of interventions that may reduce disparities or inequities. CEBC grouped 13 programs that have the potential to reduce disparities in child welfare. The Community Guide curated 12 policies and programs in education and housing that could reduce health inequity. For those interventions, The Community Guide reported subgroup findings and reviewed intervention effects by different subpopulations such as age groups and racial/ethnic groups with consideration of applicability and generalizability.

Both WWFH and MCH rated each intervention based on their potential impact on health equity and summarized research findings (including subgroup analysis). WWFH assessed a policy or program’s likely effect on disparities and assigned one of three disparity ratings: likely to decrease disparities, no impact on disparities likely, or likely to increase disparities. Like The Community Guide, WWFH analyzed subgroup findings and various intervention effects by different populations, but such information was not presented in a separate designated section of an intervention’s page. MCH incorporated a health disparity and equity impact assessment into its overall assessment of practice impact and then assigned one of four designations to each practice: cutting-edge practice, emerging practice, promising practice, or best practice. MCH also described subgroup findings and how the practice contributed to reducing health inequities.

### Transparency About Methods to Assess an Intervention’s Health Equity Impact

In general, the seven clearinghouses provided an overview of how they assess an intervention’s potential impact on health equity, but most lacked enough detail about their methods on their website to be replicated. For the clearinghouses that curated a subset of interventions, CEBC’s criteria for interventions to be included in their list was the “program must specifically target the reduction of disparities and/or disproportionality in the child welfare system either in general or for a specific case (California Evidence-Based Clearinghouse for Child Welfare, n.d.).” However, practitioners must infer how an intervention addresses disparity and disproportionality in the system based on the target population, program goals, and the research review summaries. The Community Guide described how it prioritized the social determinants of health topic and subtopics in education and housing and that it “considered health equity across all systematic reviews (The Guide to Community Preventive Services, n.d.).” However, we could not find additional information on how the interventions in the list were selected over others. Both CEBC and The Community Guide included interventions that did not have enough evidence to demonstrate actual impacts on health equity, indicating that the intervention effectiveness did not seem to inform the selection of interventions.

For clearinghouses that assigned a disparity/equity rating, we could not find clear rationale to help users understand why interventions were assigned a rating. WWFH assigned a disparity rating to an intervention “based on its characteristics (e.g., target population, mode of delivery, cultural considerations, etc.) and best available evidence related to disparities in health outcomes (What Works for Health, n.d.),” without additional information on specific criteria for each rating. MCH provided practitioners with the minimum criteria checklist to help users assess their own practices. MCH’s equity-related criteria for its highest rating designation, “Best practice,” were described as a practice that “provides evaluation data that demonstrate how it has addressed health inequities and/or systemic oppression that impact a key population (Association of Maternal & Child Health Programs, n.d.).” However, MCH did not specify the amount and quality of evidence needed to meet each designation. HomVEE clarified the definition of a subgroup and the protocol for reviewing and reporting subgroup analyses. Specifically, HomVEE reviewed replicable subgroups and reported subgroup results only from replicated subgroups to ensure “that evidence of effectiveness is not due simply to chance” (Sama-Miller et al., 2021). Blueprints and CrimeSolutions did not describe how they review subgroup analyses of individual studies.

In terms of literature sources and study design, MCH used evaluations in addition to experimental studies, including pre-post studies, testimonials, and qualitative studies to inform its equity impact review. The remaining clearinghouses did not describe the types of sources or the standards used to assess the evidence in their health equity impact review. In most cases, we can assume that subgroup analysis was based on the same literature used to determine an intervention’s effectiveness rating.

### Health Equity Population Groups of Interest

All seven clearinghouses assessed evidence by racial and ethnic groups (Table 1). Apart from CEBC, which only assessed racial and ethnic disparities in the child welfare system, clearinghouses explored various other population groups. Five clearinghouses examined an intervention’s impacts by socioeconomic status (SES) (e.g., high vs. low SES), four by level of urbanization (e.g., urban vs. rural), three by age group (e.g., older vs. younger participants), and two by gender group (e.g., male vs. female).

### Equity-Related Intervention Characteristics

Most clearinghouse considered various levels of intervention for the health equity impact review (Table 1). The list of interventions that CEBC curated included a mix of child- and family-level services and programs and child welfare agency-level staff trainings and practices. The Community Guide focused on the social determinants of health and higher-level interventions to reduce health inequities, specifically housing and education policies for community- and system-level changes. For each housing and education intervention, The Community Guide described how homelessness and educational inequities are closely related to health disparities. For the four clearinghouses that conducted health equity impact reviews for all interventions in their databases (Blueprints, CrimeSolutions, WWFH, and MCH), they included interventions with different intervention levels. Programs for individual-, family-, and community-level changes were most common. These clearinghouses also addressed some higher-level interventions: for example, school reform (Blueprints), policing practices in the justice system (CrimeSolutions), taxation and housing policies (WWFH), and system-level practices intended for system building, shifting power, and inter-sectoral collaboration (MCH). HomVEE addressed family-level interventions only.

We also examined how an intervention’s targeting approach (i.e., universal versus targeted) was considered in a clearinghouse’s disparity impact review (Table 1). CEBC explicitly curated all targeted interventions for ethnic minority children and families involved in the child welfare system. CrimeSolutions primarily had interventions that target individuals in crisis or at risk of violence, particularly offenders and victims. HomVEE assessed home visiting interventions that serve at-risk pregnant women and families with young children. The remaining four clearinghouses included a mix of universal and targeted interventions, for example, The Community Guide’s health equity intervention list included universal education interventions and housing policies exclusively for individuals with lower incomes. The four clearinghouses’ reviews of individual study findings or disparity-related rating assignments did not appear to vary by the intervention’s targeting approach.

## Discussion

The majority of the 18 clearinghouses identified in our systematic scan did not have an explicit focus on health equity. The clearinghouses that conducted health equity impact reviews varied in their definitions of equity, priority populations included, health equity review methods, and presentation of review findings. Clearinghouses used different approaches to complete health equity impact reviews and lacked transparency about their underlying methods which demonstrates that the field is in a developmental phase for this form of translational research and has yet to reach consensus on how to assess interventions for their potential effectiveness in advancing health equity. This study did not assess which approaches to conducting and communicating findings of a health impact review were most effective for user-uptake. Future research should examine the most appropriate methods and how to distill equity-focused information for practitioners. Research that examines specific gaps in the literature should also be a priority so that clearinghouses can work with researchers to focus their studies in ways that fill evidence gaps.

### A New Role for Clearinghouses: Opportunities and Barriers

Clearinghouses can be instrumental in helping practitioners understand an intervention’s potential impact on health equity. Clearinghouses have traditionally played a key role in disseminating scientific findings to help users find evidence-informed interventions to meet their needs and goals (Paulsell et al., 2017). Clearinghouses can go beyond simple assessment of average effectiveness and instead consider how interventions influence existing disparities and inequities. This move could support practitioners in their attempts to advance equity and further influence practitioners’ decision-making and policy preferences (NASEM, 2017b). In the systematic review process that some clearinghouses use for their comprehensive research synthesis, researchers have started to pay more attention to equity-focused systematic reviews, and some researchers started to conduct systematic reviews of an intervention’s equity impact (Garnett et al., 2022; Lehne & Bolte, 2017; Smith et al., 2017; Turnbull et al., 2020). However, clearinghouses are not able to conduct equity impact reviews when there is a dearth of equity-focused research, including a lack of studies that systematically analyze subgroup effects with rigor that allow for a substantial assessment of potential impact on health equity.

There is often tension between what researchers study and what information practitioners need to implement an intervention. Although practitioners typically value the relevance of local context, researchers often value methodologies that produce high internal validity, like randomized control trials, which by design reduce the nuance of local context (Alvidrez et al., 2019). Practitioners often need to consider factors outside evidence of average effectiveness, including relevance of the intervention for the focus population, community context, and service delivery system (Horne, 2017; Paulsell et al., 2017). This is especially the case for practitioners who may need to tailor an intervention to advance equity in their community without compromising effectiveness (Alvidrez et al., 2019). Researchers can strengthen the evidence base by assessing and reporting both average effects and differential effects among subgroups of an intervention to fully explain how the intervention works for various subpopulations and affects health disparities and inequities (Maden et al., 2017; Welch et al., 2022; Whitlock et al., 2017). Lastly, there are growing calls for researchers to apply methodologies and frameworks to better align their focus on equity (Chinman et al., 2017; Eslava-Schmalbach et al., 2019; McNulty et al., 2019; Shelton et al., 2021).

Clearinghouses can inform research agendas by calling attention to the need for an evidence base on the equity impact of interventions. Clearinghouses can also uplift system changes and interventions at various levels — individual, community, and systems change — to advance health equity. In addition, clearinghouses can broaden the types of evidence they assess so they are not limited by evidence produced through experimental designs or by what is published in peer reviewed journals. However, legislation that established federal clearinghouses may limit the types of interventions included and types of evidence assessed.

Although there are opportunities for clearinghouses to lead in aligning methods and better supporting practitioners who want to advance equity, they are limited by staffing and financial constraints, completing timely reviews, and understanding what level of detail practitioners need to inform their work (Burkhardt et al., 2015).

### Strengths and Limitations

This study has several strengths. The list of clearinghouses we identified included both government- and non-government-hosted clearinghouses and covered diverse topic areas addressing the social determinants of health. This captured a wide breadth of clearinghouses and their approach to equity. Another strength of this study is that we examined how clearinghouses conducted equity impact reviews from various angles, by analyzing not only clearinghouses’ approaches to communicating an intervention’s impact on equity, but also how they defined equity and considered intervention characteristics that can advance equity.

This study is not without limitations. First, despite a systematic approach to identifying clearinghouses for this study, the list was not exhaustive due to the refined search terms and research scope, emergence of tools, or their mode of dissemination for practitioners. For example, clearinghouses with state- or sector-specific focus and no explicit health tie (e.g., Ohio Evidence-based Clearinghouse, Evidence for ESSA, CASEL Program Guide) did not surface using our methods but were identified by experts who reviewed this manuscript. Second, this study relied on publicly available information from each clearinghouse’s website. Therefore, the information we collected might not fully represent the clearinghouses’ approaches to equity. Third, the comparative analysis used a qualitative review and does not preclude bias in authors’ interpretation of collected clearinghouse information. Specifically, because the authors were part of one of the selected clearinghouses and conducted a non-blind review, the interpretation was not able to be completely separated from the authors’ established definitions and values. Finally, this research paper summarizes information on the state of health equity methods among evidence clearinghouses but does not evaluate the effectiveness of these methods as this was beyond the scope of this paper. Future research will address the effectiveness of various methods for equity impact review and their communication and translation to action.

## Conclusion

This is the first study to our knowledge that examined how and the extent to which clearinghouses considered equity in their review of evidence. Clearinghouses play an important role in assessing and summarizing evidence for practitioners, but clearinghouses are not able to fully conduct health equity impact reviews until there is a sufficient underlying evidence base for the differential effects of interventions among population groups or social identities. This study found that most clearinghouses have not yet incorporated a health equity impact review and those who have are not aligned or explicit about how health equity is defined and operationalized. Advancing equity through an evidence-informed approach will require researchers to conduct more equity-focused research and clearinghouses to evolve as practice-oriented tools with health equity impact reviews based on clear and transparent underlying definitions, values, and methods.

## Supplementary Information

Below is the link to the electronic supplementary material.

Supplementary file1 (DOCX 27 KB)

## Data Availability

All data generated or analyzed during this study are included in this published article and its supplementary material.

## Footnotes

1 One clearinghouse, Home Visiting Evidence of Effectiveness (HomVEE), was initially reviewed in December 2021 with the other clearinghouses and again in 2023 during manuscript revision. The analysis for HomVEE still reflects information from December 2021.

2 Model Programs Guide by Office of Juvenile Justice Delinquency Prevention shared a database of juvenile programs with CrimeSolutions and was excluded.

3 Beginning with its 2021 annual review, HomVEE started listing which subgroups were examined in a study on the details page of each manuscript where the quality of findings was rated as moderate or high. Subgroup findings were not reported unless replicated.
