# Supplementary material for: Evidence Clearinghouses as Tools to Advance Health Equity: What We Know from a Systematic Scan
Source: Prev Sci. 2023 Mar 1;24(4):613–24. doi: 10.1007/s11121-023-01511-7 (PMC10227106; doi:10.1007/s11121-023-01511-7)
Supplement: Supplementary file 1 — Supplementary file1 (DOCX 27 KB) [file 11121_2023_1511_MOESM1_ESM.docx]

**SUPPLEMENTARY MATERIAL**

*General Information of Evidence Clearinghouses that Assign an Intervention Effectiveness Rating*

| Clearinghouse name | Topic area(s) | Host organization | Search filters (that users can search interventions with for an equity-enhancing goal) |
| --- | --- | --- | --- |
| **Governmental Clearinghouses (10)** | | | |
| [AmeriCorps Evidence Exchange](https://americorps.gov/about/our-impact/evidence-exchange) | National service, social innovation, civic engagement, and volunteering | AmeriCorps | None |
| [CrimeSolutions](https://www.crimesolutions.gov/) | Criminal justice | U.S. Department of Justice, the National Institute of Justice | Race/ethnicity, LGBTQ, and geography |
| [Evidence-Based Cancer Control Programs (formerly Research-Tested Intervention Programs)](https://rtips.cancer.gov/rtips/index.do) | Cancer control interventions and programs including topics of cancer screening, HPV vaccination, obesity, diet/nutrition, physical activity, sun safety, supportive care, and tobacco control | U.S. Department of Health & Human Services, National Cancer Institute | Race/ethnicity, community type (urban/suburban/rural), and age |
| [Home Visiting Evidence of Effectiveness (HomVEE)](https://homvee.acf.hhs.gov/) | Home visiting | U.S. Department of Health & Human Services, Administration for Children & Families; contract with Mathematica Policy Research | Tribal |
| [Model Programs Guide](https://ojjdp.ojp.gov/model-programs-guide/home) | Juvenile justice system and delinquency | U.S. Department of Justice, Office of Juvenile Justice and Delinquency Prevention | Racial and ethnic minorities, tribal youth, youth with disabilities, foster care/child welfare system involved youth, and age |
| [Pathways to Work Evidence Clearinghouse](https://pathwaystowork.acf.hhs.gov/) | Employment | U.S. Department of Health and Human Services, Administration for Children & Families, The Office of Planning, Research & Evaluation | Urban or rural settings, education level, employment status, single or noncustodial parents, sex, and employment barriers (including disabilities, mental health issues, and justice system involvement) |
| [Rural Health Models and Innovations](https://www.ruralhealthinfo.org/project-examples) | Rural health | Federal Office of Rural Health Policy | Racial and ethnic groups, limited English proficiency, poverty, uninsured and underinsured, etc. |
| [The Guide to Community Preventive Services (The Community Guide)](https://www.thecommunityguide.org/) | Public health including the social determinants of health | U.S. Department of Health and Human Services, Centers for Disease Control and Prevention, The Community Preventive Services Task Force | Health equity |
| [Title IV-E Prevention Services Clearinghouse](https://preventionservices.abtsites.com/) | Mental health, substance abuse, parenting, kinship navigator programs | U.S. Department of Health and Human Services, Administration for Children and Families | None |
| [What Works Clearinghouse](https://ies.ed.gov/ncee/wwc/) | Education | U.S. Department of Education, Institute of Education Sciences | Children and youth with disabilities, and English learners |
| **Academic/Nonprofit Clearinghouses (8)** | | | |
| [Blueprints for Healthy Youth Development](https://www.blueprintsprograms.org/) | Prevention of problem behavior and promotion of healthy youth development across multiple fields (criminal justice, child welfare, public health, mental health, education, labor/employment). | University of Colorado Boulder, Institute of Behavioral Science, Prevention Science Program; currently funded by Arnold Ventures (formerly the Laura and John Arnold Foundation) | Race/ethnicity, family risk factors (e.g., low socioeconomic status, overcrowded living situation), and neighborhood risk factors (e.g., community disorganization, extreme economic disadvantage, perceived racism/ discrimination, and mobility) |
| [California Evidence-Based Clearinghouse for Child Welfare (CEBC)](http://www.cebc4cw.org/) | Child welfare | California Department of Social Services’ Office of Child Abuse Prevention | Reducing racial disparity and disproportionality in child welfare |
| [Clearinghouse for Military Family Readiness (Continuum of Evidence)](https://www.continuum.militaryfamilies.psu.edu/) | Family and mental health | Pennsylvania State University, Social Science Research Institute; funded by the U.S. Department of Defense | Individuals with disabilities, and older adults |
| [MCHbest database](https://www.mchevidence.org/tools/strategies/) | Maternal and child health | Georgetown University, National Center for Education in Maternal and Child Health, MCH Evidence Center; funded by the U.S. Department of Health and Human Services, Health Resources and Services Administration | None |
| [[MCH Innovations Database (formerly Innovation Station Database)](https://amchp.org/mch-innovations-database/)](https://amchp.org/mch-innovations-database/) | Maternal and child health | Association of Maternal & Child Health Programs (AMCHP); funded by the Health Resources and Services Administration (HRSA) of the U.S. Department of Health and Human Services (HHS) | Health equity, access to health care/insurance, and rural |
| [Promising Practices](https://cdc.thehcn.net/index.php?module=promisepractice&controller=index&action=index) | Health, community, economy, education, environmental health | Conduent Healthy Communities Institute (Conduent HCI) | Women, older adults, racial/ethnic minorities, and rural areas |
| [Social Programs That Work](https://evidencebasedprograms.org/) | Social programs across various social policy areas | Arnold Ventures (formerly the Laura and John Arnold Foundation) | None |
| [What Works for Health (WWFH)](https://www.countyhealthrankings.org/take-action-to-improve-health/what-works-for-health) | Public health including the social determinants of health | University of Wisconsin Madison, Population Health Institute; funded by the Robert Wood Johnson Foundation and the Wisconsin Partnership Program | Access to care |
